# Call for the Standardization of Opioid Related Terminologies

**DOI:** 10.31480/2330-4871/196

**Published:** 2025-01-17

**Authors:** Renyu Liu, John Grothusen, Gordon Barr

**Affiliations:** Department of Anesthesiology and Critical Care, Perelman School of Medicine at the University of Pennsylvania, USA

As we continue to collaborate and communicate with scientists and clinicians in various subspecialties who work on opioids, opioid receptor pharmacology, pain management, and opioid addiction management, we believe that there is urgent need to standardize terminologies related to opioid and opioid crisis management. It is often difficult to communicate with the public since the terminology used or the names of medications in clinical practice can appear very complicated and confusing to non-specialists.

The complexity of opioids and their opioid receptors make it very difficult to understand even to medical professionals. Classically there are three typical opioid receptors, namely mu (MOR), delta (DOR), and kappa (KOR) opioid receptors. Each category of receptor has specific natural (endogenous ligand) or man-made (drug) agonists that fully activate the receptor, partial agonists that partially activate the receptor or its specific signaling pathway, and antagonists that can deactivate the receptor in the presence of the agonist. Natural and man-made compounds may act differently from each other in each receptor type. Some of compounds may activate MOR, but only partially activate or even antagonize the other opioid receptors, for example KOR. Even in the same receptor, a compound may have different receptor activation or deactivation specific molecular signaling pathways, namely the G protein pathway and the beta-Arrestin pathway. Thus at minimum the consequences of a drug or endogenous ligand targeting a receptor depends on the receptor type, action at that receptor and subsequent signaling pathway.

Therefore, it should not be surprising that opioid compounds may have a variety of different pharmacological properties and physiological consequences when they are used in clinical practice. Unless someone has a understanding of the molecular pharmacology of opioid receptors, it is often difficult for them to comprehend the complexity of each category of medication since some terminologies are based on specific molecular activation pathways. For example, a biased ligand is one that can activate one signaling pathway, of the receptor, either G-protein or beta-Arrestin, but may have no or minimal effects on the other signaling pathway. It is often difficult to find common ground for communication of opioid actions, especially with people in the non-specialist public. In the lay media, the term opioids, though commonly used, includes a large category of medications that have very different consequences when used for long periods of time. When we talk about an opioid crisis, we are talking about a small number of opioid compounds that are highly addictive and can induce respiratory depression and cardiac arrest. There are terminologies related to opioids, such as opiate and narcotics. While all three terminologies are potentially interchangeable, there are subtle medical, pharmaceutical, and legal differences. Such subtle differences are not generally well appreciated by the public. The term opiate generally refers to natural substances derived from the opium poppy plant; and the term opioid refers to synthetic chemicals that act on opioid receptors, usually receptor agonists for medical field. The term narcotic is a broader term that includes both opiates and opioids that commonly appear in legal contexts. Even the same opiate or opioid related chemicals might have different pharmaceutical, medical or street names that are not necessary mutually recognized by clinicians, general physicians, law enforcement or lay people.

In a recent group discussion with experts working on opioid pharmacology and opioid addiction therapy, the notion of the opioid blockade effects of methadone emerged during the discussion and resulted in a hot discussion as to whether the “blockade” should still be used since it means differently in different field. Upon literature exploration, we found that the first paper discussing “narcotic blockade” was published in 1966 [1]. This paper concluded that “A stable blockade against the narcotic effects of heroin can be maintained by a single daily oral dose of methadone. Blockade is established by gradual increase in dosage to a stabilization level” [1]. This overly simplistic explanation can be excused since this paper was originally published in 1966 and the opioid receptor was not discovered until 1973 [2]. Following the discovery of the receptor, the concept of blockade in pharmacology generally means something that can block or antagonize the receptor from the activation of the receptor agonist. Similar to morphine, methadone is a full MOR agonist. Therefore, in the modern understanding of opioid pharmacology, it is confusing if we still use the notion of opioid blockade effects from methadone. However, such notions continue appearing in scientific collaborative discussions and in recent scientific literature [3–5]. The outdated concept of “narcotic blockade” is even more confusing for buprenorphine since buprenorphine is a partial agonist on MOR and can act as a partial agonist (potentially blockage effects) for other full agonist [6–8]. Therefore, we would highly suggest not using the word “blockade” in opioid behavior modification to avoid confusion.

In conclusion, multi-disciplinary team should be formed to come out a consensus of the standard terminologies for opioids for both the science community and for public educational purposes. If you are interested in joining such discussion, please feel free to reach out to Dr. Renyu Liu: RenYu.Liu@pennmedicine.upenn.edu
